# Supplementary material for: Edible Fruits from the Ecuadorian Amazon: Ethnobotany, Physicochemical Characteristics, and Bioactive Components
Source: Plants (Basel). 2023 Oct 21;12(20):3635. doi: 10.3390/plants12203635 (PMC10610027; doi:10.3390/plants12203635)
Supplement: Supplementary file 1 [file plants-12-03635-s001.zip › plants-2634615-supplementary.pdf]

Supplementary Material

Table S1. Ethnobotanical information on edible fruits of the Ecuadorian Amazon

Nº	Scientific name	Vernacular name	Location reported in Ecuador (Province)	Use	Part of fruit used	Consumption form	Origin*	Source
**ARECACEAE**								
**1**	*Aphandra natalia* (Balslev & A.J. Hend.) Barfod	Tagua, marfil vegetal, Chilli, Chilli-punschu, Chiri'si, Fibra, Kinchuk, Kintiuk Sili, Tindiuqui, Tintiuk, Wamowe (Ecuador) and Piassaba, Piassaba, Piassava	Pastaza	Food, materials	Pulp, seed	Mesocarp is eaten like fresh fruit or after boiling, the immature endosperm is consumed as a beverage and the seed is used like vegetable ivory (local name "Tagua") to manufacture buttons and carvings	Native, cultivated	[1–3]
**2**	*Attalea colenda* (O.F. Cook) Balslev & A.J. Hend	Palma real	Amazon	Food, medicine	NR		Native, cultivated	[4]
**3**	*Bactris gasipaes* Kunth	Chonta, chontaduro, peach palm, persikopalm o pupunh, hontaduro, pejiballe, acana o pupunha<br>Chonta, Chonta dura, Chontaduro, Chonta palm, Palmito, Puka chunta, Shalin chunta	Orellana	Cultural, food, materials, medicine	Pulp, seed	Roasted or cooked fruit, flour, non-alcoholic or fermented alcoholic beverage called chicha.<br>Juices, canned goods, jams, and preserves, animal feed, hunting and fishing bait, and fish poison mixed with Clibadium surinamense leaves. Its oil is used as a cosmetic, lubricant, and soap	Introduced, cultivated	[5–8]

						for human consumption. The seed is used in handicrafts.		
**4**	*Bactris maraja* Mart.	Chontilla	Amazon	Food, medicine	Whole fruit, seed		Native	[4]
**5**	*Cocos nucifera* L.	Coco	Orellana, Sucumbios	Medicine	Pulp, Seed	Candies, juices, coconut milk, coconut water	Introduced, cultivated	[4,5]
**6**	*Desmoncus orthacanthos* Mart.	Vara waska, tu'tu , yeyedokame	Amazon	Feed, Medicine	Whole fruit	Fresh fruit	Native	[4]
**7**	*Euterpe oleracea* Mart.	Asaí, azaí, huasaí, palma murrapo, naidí, or generally acai	Amazon	Food, medicine	Pulp	Fresh fruit	Native	[5]
**8**	*Mauritia flexuosa* L.f.	Aguaje, buriti, morete, moriche o muriti, canangucha, caranday-guazu, palma real, ite palm	Napo, Orellana, Pastaza, Sucumbios	Cultural, food, feed, materials, medicine	Pulp, seed	Fresh and cooked fruit, for oil extraction, preparation of non-fermented and fermented beverages, creams, jellies and jams.	Native, cultivated	[9–11]
**9**	*Mauritiella armata* (Mart.) Burret	Wichilla morete	Amazon	Feed, food medicine	Whole fruit	Fresh fruit	Native	[4]
**10**	*Oenocarpus bataua* Mart.	Bataua, chapil, patawa, patauá, seje, mil pesos, ungurahua o ungurahui	Napo, Sucumbios	Cultural, food, materials, medicine	Pulp, pell, seed	Fresh or cooked fruit, in iced preparations, unfermented and fermented beverages such as chicha, for the extraction of oil, which is used as a hair tonic.	Native, cultivated	[4–6,12,13]
**RUTACEAE**								
**11**	*Citrus x paradisi* Macfad	Pomelo	Orellana, Sucumbios	Food	Pulp	Fresh fruit	Introduced, cultivated	[5]
**12**	*Citrus aurantifolia* Swingle	Limón	Amazon	Medicine	Pulp	Preparations whith juice and other specie	Introduced, cultivated	[14]

**13**	*Citrus reticulata* Blanco	Mandarina	Orellana, Sucumbios	Food	Pulp	Fresh fruit	Introduced, cultivated	[5]
**14**	*Citrus x aurantium* L.	Limon	Orellana, Sucumbios	Food	Pulp	Fresh fruit	Introduced, cultivated	[5]
**15**	*Citrus x sinensis* (L.) Osbeck	Naranja	Orellana, Sucumbios	Food	Pulp	Fresh fruit	Introduced, cultivated	[5]
**MALVACEAE**								
**16**	*Gossypium barbadense* L.	Algodon, Uruch	Amazon	Medicine	Whole fruit	Roasted fruit extract	Native, cultivated	[14]
**17**	*Matisia cordata* Bonpl.	Zapote	Orellana, Sucumbios	Feed, food	Pulp	Fresh fruit	Native, cultivated	[5]
**18**	*Theobroma bicolor* Bonpl.	Cacao blanco, árbol patas, sus semillas conocidas como patas-muyo	Napo, Orellana, Sucumbios	Food	Pulp, seed	Fresh pulp, roasted seed	Introduced, cultivated	[8,15]
**19**	*Theobroma grandiflorum* (Willd. ex Spreng.) K. Schum.	Copoazú	Amazon	Food, medicine	Pulp, seed	Fres fruit, like cosmetic, chocolate, beverage, liquor, and food	Introduced, cultivated	[5]
**20**	*Theobroma cacao* L		Orellana, Zamora Chinchipe	Food, materials	Pulp, seed	Fresh fruit, chocolate	Native, cultivated	[16,17]
**MYRTACEAE**								
**21**	*Eugenia malaccensis* L	Pomarrosa	Orellana, Sucumbios	Food	Whole fruit	Fresh fruit	Introduced, cultivated	[5]
**22**	*Eugenia stipitate* McVaugh	pa, Quince, Amazonic guava,	Amazon	Food, medicine	Pulp	Fresh fruit, juice	Native, cultivated	[5,18]
**23**	*Myrciaria dubia* (Kunth) McVaugh	Camu-camu, caçari, arazá de agua, guayabo, guayabito, guapuro blanco	Amazon	Feed, food, medicine	pulp, pell	Extract, juices, nectars, yogurts, ice cream, jams, alcoholic beverages or soft drinks	Native	[5,10]

**24**	*Psidium guayava* Raddi	Kirim, guayaba	Morona Santiago, Orellana, Pastaza, Sucumbios	Food, medicine	Whole fruit	Fresh fruit, decoction	Native, cultivated	[5]
**MORACEAE**								
**25**	*Artocarpus altilis* (Parkinson) Fosberg	Fruta de pan	Orellana, Sucumbios	Food	Seed	Fresh fruit	Cultivated	[5]
**26**	*Artocarpus heterophyllus* Lam.	Fruta de jaca	Orellana, Sucumbios	Food	Pulp	Fresh fruit		[5]
**27**	*Ficus carica* L.	Higo	Orellana, Sucumbios	Food	Whole fruit	Fresh fruit	Introduced, cultivated	[5]
**PASSIFLORACEAE**								
**28**	*Passiflora edulis* Sims.	Maracuyá	Orellana, Sucumbios	Food	Pulp	Fresh fruit	Introduced, cultivated	[5]
**29**	*Passiflora incarnata* L.	Granadilla silvestre	Orellana, Sucumbios	Food	Pulp	Fresh fruit		[5]
**30**	*Passiflora quadrangularis* L.	Badea	Orellana, Sucumbios	Food	Pulp	Fresh fruit	Native	[5]
**LECYTHIDACEAE**								
**31**	*Grias neuberthii* J.F. Macbr.	Pitón	Orellana	Food	Pulp	Fresh fruit or rosted	Native, cultivated	[8]
**32**	*Gustavia macarenensis* Philipson	Alan paso, Inaco, Inak, Passo, Sachu pasu y, en el presente estudio, Pasu	Napo, Orellana, Sucumbios	Food, medicine	Pulp, pell, seed	Juice, and oil	Native	[5,8,13, 19]
**RUBIACEAE**								
**33**	*Borojoa patinoi* Cuatrec.	Borojo	Orellana, Sucumbios	Food	Pulp	Fresh fruit, energizing, jam and vinaigrette	Native	[5]
**34**	*Morinda citrifolia* L.	Noni	Amazon	Food	Pulp	Fresh fruit		[5]
**SAPINDACEAE**								
**35**	*Nephelium lappaceum* Poir.	Achotillo, Rambután	Orellana, Sucumbios	Food	Pulp	Fresh fruit	Introduced	[5]
**SOLANACEAE**								

**36**	*Solanum quitoense var. palora* Lam.	Naranjilla	Napo, Orellana, Sucumbios	Food	Whole fruit	Fresh fruit, juice	Native, cultivated	[18,20]
**37**	*Solanum sessiliflorum* Dunal	Cocona	Amazon	Food, medicine	pulp, pell	Fresh fruit	Native, cultivated	[5]
**ANNONACEA**								
**38**	*Annona cherimola* Mill.	Chirimoya	Orellana, Sucumbios	Food	Pulp	Fresh fruit	Native, cultivated	[5]
**39**	*Annona muricata* L.	Guanábana	Orellana, Sucumbios	Food	Pulp	Fresh fruit	Native, cultivated	[5]
**BROMELIACEAE**								
**40**	*Ananas comosus* (L.) Merr.	Piña	Orellana, Sucumbios	Food	Pulp	Fresh fruit	Introduced, cultivated	[5]
**ANACARDIACEAE**								
**41**	*Anacardium occidentale* L.	Marañon	Orellana, Sucumbios	Food	Pulp, seed	Fresh fruit, juice	Introduced	[5]
**52**	*Spondias mombin* L.	Hobo	Orellana, Sucumbios	Food, medicine		Fresh fruit	Native, cultivated	[5]
**OXALIDACEAE**								
**43**	*Averrhoa carambola* L.	Carambola	Orellana, Sucumbios	Food	Whole fruit	Fresh fruit	Introduced, cultivated	[5]
**CARICACEAE**								
**44**	*Carica papaya* L.	Papaya	Orellana, Sucumbios	Food	Pulp	Fresh fruit	Native, cultivated	[21]
**BURSERACEAE**								
**45**	*Dacryodes peruviana* (Loes.) H.J. Lam	Copal, copal comestible, anime, wigonkawe	Morona Santiago, Napo, Pastaza, Zamora Chinchipe	Feed, food	Whole fruit	Fresh fruit, feed for monkeys, birds	Native	[22]
**CACTACEAE**								

**46**	*Hylocereus megalanthus* (K. Schum. ex Vaupel) Ralf Bauer	Yellow pitahaya	Morona Santiago, Orellana, Sucumbíos	Food	Pulp	Fresh fruit	Introduced, cultivated	[5,23]
**FABACEAE**								
**47**	*Inga edulis* Mart.	Guaba	Orellana, Sucumbios	Food	Arilo	Fresh fruit	Native, cultivated	[5]
**ACHARIACEAE**								
**48**	*Mayna yasuniana* Á.J. Pérez, Liesner & D. Santam.	Huaorani: dakatoguawe, numayabo, yepenemoncamo	Orellana	Food, medicine	Pulp	Fresh fruit	47	[24]
**NYCTAGINACEAE**								
49	*Neea sp.* Ruiz & Pav.	NA	Orellana	Medicine	Whole fruit	Fruit chewing to prevent cavities	Native	[25]
**LAURACEAE**								
**50**	*Persea americana* Mill.	Aguacate	Orellana, Sucumbios	Food	Pulp	Fresh fruit	Native, cultivated	[5]
**PHYLLANTHACEAE**								
**51**	*Phyllanthus acidus* (L.) Skeels.	Grosella	Orellana, Sucumbios	Food		Fresh fruit	Introduced, cultivated	[5]
**EUPHORBIACEAE**								
**52**	*Plukenetia volubilis* L.	Sacha inchi, sacha yachi, sacha yuchi, sacha yuchiqui, yuchi, sampannankii, suwaa, correa, amauebe, amui-o, maní de arbol, maní del monte, maní estrella, nuez del inca	Amazon	Feed, food, materials	Seed	Roasted seeds, salted or covered in chocolate, pressed for oil, or ground to a buttery substance. Proces products like protein powder or flour, nanoparticles and biofuel		[26,27]
**URTICACEAE**								

**53**	*Pourouma cecropiifolia* Mart.	Uva de árbol	Orellana, Sucumbios	Food	Whole fruit	Fresh fruit	Native, cultivated	[5]
**SAPOTACEAE**								
**54**	*Pouteria caimito* (Ruiz & Pav.) Radlk.	Caimito, avío	Orellana, Sucumbios	Food	Pulp	Fresh fruit	Native, cultivated	[5,8]
**ROSACEAE**								
**55**	*Prunus cerasifera* Ehrh.	Ciruelo	Orellana, Sucumbios	Food	Whole fruit	Fresh fruit	Introduced, cultivated	[5]

* The origin of the species was described according to the Encyclopedia of useful plants of Ecuador
The families were identified according to The Tropicos database (https://www.tropicos.org/home)
NR. No reported

1. Viafara, D.; Abreu-Naranjo, R.; Alvarez-Suarez, J.M.; Reyes-Mera, J.J.; Barreno-Ayala, M. Chemical Characterisation and Antioxidant Activity of Aphandra Natalia Mesocarp and Its Oil from the Amazon Region of Ecuador. *Journal of Food Measurement and Characterization* **2018**, *12*, 2835–2843, doi:10.1007/s11694-018-9898-x.
2. Kronborg, M.; Grández, C.A.; Ferreira, E.; Balslev, H. Aphandra Natalia (Arecaceae)-a Little Known Source of Piassaba Fibers from the Western Amazon. *Rev Peru Biol* **2008**, *15*, 103–113.
3. Pedersen, H.B. Production and Harvest of Fibers from Aphandra Natalia (Palmae) in Ecuador. *For Ecol Manage* **1996**, *80*, 155–161, doi:https://doi.org/10.1016/0378-1127(95)03632-6.
4. Paniagua-Zambrana, N.; Cámara-Leret, R.; Macía, M.J. Patterns of Medicinal Use of Palms Across Northwestern South America. *The Botanical Review* **2015**, *81*, 317–415, doi:10.1007/s12229-015-9155-5.
5. Vargas-Tierras, Y.B.; Prado-Beltran, J.K.; Nicolalde-Cruz, J.R.; Casanoves, F.; Virginio-Filho, E.D.; Viera-Arroyo, W.F. Characterization and Role of Amazonian Fruit Crops in Family Farms in the Provinces of Sucumbios and Orellana (Ecuador). *REVISTA CORPOICA-CIENCIA Y TECNOLOGIA AGROPECUARIA* **2018**, *19*, 501–516, doi:10.21930/rcta.vol19_num3_art:812.
6. Jaramillo-Vivanco, T.; Balslev, H.; Montúfar, R.; Cámara, R.M.; Giampieri, F.; Battino, M.; Cámara, M.; Alvarez-Suarez, J.M. Three Amazonian Palms as Underestimated and Little-Known Sources of Nutrients, Bioactive Compounds and Edible Insects. *Food Chem* **2022**, *372*, 131273, doi:https://doi.org/10.1016/j.foodchem.2021.131273.

7. González-Jaramillo, N.; Bailon-Moscoso, N.; Duarte-Casar, R.; Romero-Benavides, J.C. Peach Palm (Bactris Gasipaes Kunth.): Ancestral Tropical Staple with Future Potential. *Plants* **2022**, *11*, doi:10.3390/plants11223134.
8. Innerhofer, S.; Bernhardt, K.-G. Ethnobotanic Garden Design in the Ecuadorian Amazon. *Biodivers Conserv* **2011**, *20*, 429–439, doi:10.1007/s10531-010-9984-9.
9. Abreu-Naranjo, R.; Paredes-Moreta, J.G.; Granda-Albuja, G.; Iturralde, G.; González-Paramás, A.M.; Alvarez-Suarez, J.M. Bioactive Compounds, Phenolic Profile, Antioxidant Capacity and Effectiveness against Lipid Peroxidation of Cell Membranes of Mauritia Flexuosa L. Fruit Extracts from Three Biomes in the Ecuadorian Amazon. *Heliyon* **2020**, *6*, doi:10.1016/j.heliyon.2020.e05211.
10. Arellano-Acuna, E.; Rojas-Zavaleta, I.; Maria Paucar-Menacho, L. Camu-Camu (Myrciaria Dubia): Tropical Fruit of Excellent Functional That Help to Improve the Quality of Life. *SCIENTIA AGROPECUARIA* **2016**, *7*, 433–443, doi:10.17268/sci.agropecu.2016.04.08.
11. Rivera, M.; Ramos, M.; Silva, M.; Briceno, J.; Alvarez, M. Effect of the Temperature Prior to Extraction on the Yield and Fatty Acid Profile of Morete Oil (Mauritia Flexuosa L.F.). *GRANJA-REVISTA DE CIENCIAS DE LA VIDA* **2022**, *35*, 98–111, doi:10.17163/lgr.n35.2022.08.
12. Miller, C. Fruit Production of the Ungurahua Palm (Oenocarpus Bataua Subsp Bataua, Arecaceae) in an Indigenous Managed Reserve. *Econ Bot* **2002**, *56*, 165–176, doi:10.1663/0013-0001(2002)056[0165:FPOTUP]2.0.CO;2.
13. Méndez-Durazno, C.; Cisneros-Perez, P.A.; Loja-Ojeda, B.A.; Monge-Sevilla, R.; Romero-Estévez, D.; Fernández, L.; Espinoza-Montero, P.J. Antioxidant Capacity through Electrochemical Methods and Chemical Composition of Oenocarpus Bataua and Gustavia Macarenensis from the Ecuadorian Amazon. *Antioxidants* **2023**, *12*, doi:10.3390/antiox12020318.
14. Giovannini, P. Medicinal Plants of the Achuar (Jivaro) of Amazonian Ecuador: Ethnobotanical Survey and Comparison with Other Amazonian Pharmacopoeias. *J Ethnopharmacol* **2015**, *164*, 78–88, doi:https://doi.org/10.1016/j.jep.2015.01.038.
15. Ponce-Sanchez, J.; Zurita-Benavides, M.G.; Penuela, M.C. Reproductive Ecology of White Cacao (Theobroma Bicolor Humb. & Bonpl.) in Ecuador, Western Amazonia: Floral Visitors and the Impact of Fungus and Mistletoe on Fruit Production. *BRAZILIAN JOURNAL OF BOTANY* **2021**, *44*, 479–489, doi:10.1007/s40415-021-00709-9.
16. Sanchez-Capa, M.; Viteri-Sanchez, S.; Burbano-Cachiguango, A.; Abril-Donoso, M.; Vargas-Tierras, T.; Suarez-Cedillo, S.; Mestanza-Ramón, C. New Characteristics in the Fermentation Process of Cocoa (Theobroma Cacao L.) “Super Arbol” in La Joya de Los Sachas, Ecuador. *Sustainability* **2022**, *14*, doi:10.3390/su14137564.
17. Herrera, R.; Vasquez, S.C.; Granja, F.; Molina-Muller, M.; Capa-Morocho, M.; Guaman, A.O. Interaction of N, P and K on Soil Characteristics, Growth and Quality of Cocoa Sprouts and Fruits in Ecuadorian Amazon. *BIOAGRO* **2022**, *34*, 277–288, doi:10.51372/bioagro343.7.
18. Llerena, W.; Samaniego, I.; Angós, I.; Brito, B.; Ortiz, B.; Carrillo, W. Biocompounds Content Prediction in Ecuadorian Fruits Using a Mathematical Model. *Foods* **2019**, *8*, doi:10.3390/foods8080284.

19. Mera, J.J.R.; Abreu-Naranjo, R.; Alvarez-Suarez, J.M.; Viafara, D. Chemical Characterization, Fatty Acid Profile and Antioxidant Activity of Gustavia Macarenensis Fruit Mesocarp and Its Oil from the Amazonian Region of Ecuador as an Unconventional Source of Vegetable Oil. *GRASAS Y ACEITES* **2019**, *70*, doi:10.3989/gya.0569181.
20. Navarrete, A.T.; Burgos, J.C. V; Quintana, Y.G.; Crespo, Y.A. Partial Chemical Composition, and Morphometric and Sensory Characteristics of Organically and Conventionally Produced Fruits of Naranjilla (Solanum Quitoense Var. Palora) in the Wamani Community, Ecuadorian Amazon. *Interciencia* **2018**, *43*, 115–119.
21. Tierras, Y.B. V; Martinez, A.E.D.; Yepez, C.D.C.; Jaramillo, L.A.T.; Arroyo, W.F. V Comparison of Fruit Quality Traits of Papaya (Carica Papaya L.) Genotypes from Shushufindi and La Joya de Los Sachas, Ecuador. *REVISTA CORPOICA-CIENCIA Y TECNOLOGIA AGROPECUARIA* **2021**, *22*, doi:10.21930/rcta.vol22_num1_art:1930.
22. Valarezo, E.; Ojeda-Riascos, S.; Cartuche, L.; Andrade-Gonzalez, N.; Gonzalez-Sanchez, I.; Meneses, M.A. Extraction and Study of the Essential Oil of Copal (Dacryodes Peruviana), an Amazonian Fruit with the Highest Yield Worldwide. *PLANTS-BASEL* **2020**, *9*, doi:10.3390/plants9121658.
23. Vargas-Tierras, Y.; Diaz, A.; Caicedo, C.; Macas, J.; Suarez-Tapia, A.; Viera, W. Benefits of Legume Species in an Agroforestry Production System of Yellow Pitahaya in the Ecuadorian Amazon. *Sustainability* **2021**, *13*, doi:10.3390/su13169261.
24. Santamaría-Aguilar, D.; Pérez, Á.J.; Liesner, R.L. Mayna Yasuniana (Achariaceae), Una Especie Nueva Para El Ecuador. *Neotrop Biodivers* **2017**, *3*, 50–56, doi:10.1080/23766808.2017.1283902.
25. Weckmüller, H.; Barriocanal, C.; Maneja, R.; Boada, M. Factors Affecting Traditional Medicinal Plant Knowledge of the Waorani, Ecuador. *Sustainability* **2019**, *11*, doi:10.3390/su11164460.
26. Kodahl, N. Sacha Inchi (Plukenetia Volubilis L.)-from Lost Crop of the Incas to Part of the Solution to Global Challenges? *Planta* **2020**, *251*, doi:10.1007/s00425-020-03377-3.
27. Fukalova-Fukalova, T.; Castillo, J.; Parreño, K.; Gaibor, M.; Londoño-Larrea, P. Preliminary Studies of Performance and Lipid Profiles of Ecuadorian P. Volubilis L. as Contribution to Agricultural Innovation. In Proceedings of the Smart Innovation, Systems and Technologies; 2022; Vol. 252, pp. 151–163.
